# The Validity of the Screen for Child Anxiety Related Emotional Disorders Revised (SCARED-R) Scale and Sub-Scales in Swedish Youth

**DOI:** 10.1007/s10578-017-0746-8

**Published:** 2017-07-29

**Authors:** Tord Ivarsson, Gudmundur Skarphedinsson, Markus Andersson, Håkan Jarbin

**Affiliations:** 1 The Centre for Child and Adolescent Mental Health, Postboks 4623 Nydalen, 0405 Oslo, Norway; 2 The Faculty of Psychology, University of Iceland, Reykjavík, Iceland; 3 Department of Clinical Sciences, Child and Adolescent Psychiatry, Lund University, Lund, Sweden; 4 Region Halland, Sweden. BUP, HSH, SE-301 85 Halmstad, Sweden

**Keywords:** Self-rating scale, Parent rating scale, Anxiety disorder, Concurrent validity, Discriminant validity, LEAD diagnosis

## Abstract

We evaluated the clinical utility of the Swedish SCARED-R in child- and adolescent psychiatric outpatients (n = 239) and validated it against Longitudinal Expert All Data (LEAD) DSM IV diagnoses based on the Children’s Schedule for Affective Disorders and Schizophrenia (KSADS) and subsequent clinical work-up and treatment outcome. The SCARED-R total score and subscales had acceptable sensitivity/specificity for child and parent reports for cut-offs based on Receiver Operating Characteristics (ROC) curves, with mostly moderate area under the curve. Sensitivity ranged from 75% (parent rated social anxiety) to 79% [child rated Generalized Anxiety Disorder (GAD)]. Specificity, ranged from 60% for child-rated GAD to 88% for parent rated social anxiety. Parent-child agreement was moderate, and each informant provided unique information contributing to most diagnoses. In conclusion, the SCARED-R is useful for screening anxiety symptoms in clinical populations. However, it cannot replace interview based diagnoses, nor is it adequate to use just one informant.

## Introduction

Anxiety disorders are prevalent paediatric mental disorders [1, 2]. In the short-term they are stable [3], and in the long-term they show both homo-typic [4] and hetero-typic continuity [5]. In the latter respect, childhood and adolescent anxiety disorders confer a higher risk of both depression and somatic illness, but also of future drug use [5]. Furthermore, anxiety disorders have a negative impact on various domains of functioning, e.g. at school, in social life, and in family relations [6].

Moreover, many types of burdens are associated with anxiety disorders, including increased costs associated with the illness both due to the child staying away from school and due to parents’ leave from work [7]. Some of these effects are found even in long-term follow-up in adulthood, e.g. lower income, and difficulties in social relationships [5].

Despite these disorders’ high prevalence in the general population, they are less common in child psychiatric care than their proportion in the general population would imply. Referrals to the specialized child- and adolescent psychiatry (CAP) services are greater for externalizing and disruptive disorders than for anxiety disorders. Gren Landell [8] found, for example, that just one out of five children with social anxiety disorder in a high school study had a CAP contact and Hansen [9] noted in a CAP outpatient sample that about half of those with a KSADS anxiety disorder at intake had not been referred because of anxiety (see as well [10, 11]). Similarly, Heyman [12] found in an epidemiological study 25 children with OCD (0.25% of all children 6–17 years of age), and only three of them were known within the specialized CAP services, although a good third had consulted their general practitioner. Thus, there is a need for better screens for anxiety symptoms to be used in primary care and in the school health care.

Another problem is that children with anxiety disorders do not seek help themselves like adults do. They are dependent on parents or teachers to recognize the symptoms and the need. Thus, there is a question of how to evaluate and compare the information an informant provides, i.e., which information, when applied in a scale, is best fitted for aiding the diagnostic process [13]. There are several challenges associated with such an evaluation. With regard to parental reports of the symptoms of anxiety disorders, those that are overt, i.e. the disorders’ behavioural manifestations, can be observed by the parent and may be easier to rate [14]. In these respects, the challenge concerns the interpretation of at least some of these overt behaviours. The interpretation is dependent on an understanding of the motivation underlying a behaviour or an adequate attribution of the cause of a behaviour [14]: is a refusal to go to school due to anxiety and an avoidance, or an expression of oppositionality with a wish to stay home and, for example, do more pleasurable activities? Thus, the level of parental insight into the child’s thoughts and feelings are crucial, and this is even more important about symptoms that are covert. They need to be reported to, or told the parent, and then translated to the items in the parent scale.

Children are commonly regarded as the best informant on subjective aspects of the disorder [15]. However, the “mapping” of these symptoms, i.e., reported by the child on a child scale is not without problems. In younger children or developmentally delayed or disordered children, a lack of introspective capacities and language skills may compromise the reliability of these assessments. Moreover, children may be less exact with describing impairment, duration, and intensity/severity.

The use of multiple informants, including both a children’s and a parental symptom assessment have, however, but a low to moderate agreement [15, 16]. Thus, we are posed with the problems on how to combine the information. In general, children tend to report more severe anxiety levels than parents do [15, 17].

Theses discrepancies in self- and parental reports makes it important to evaluate the information from different informants on a disorder specific level as the disorders vary with regard to how much the subjective perspective is central, and to which extent behavioural manifestations are core features.

Several self- and parent rating scales for anxiety symptoms have been constructed, and need to be adjusted to the changing perspectives as the diagnostic systems evolve. Recently, Muris and an expert group published an anxiety scale developed for the DSM-5 [18–20]. The Screen for Child Anxiety Related Emotional Disorders which is evaluated in the paper and that was published by Birmaher [21] in 1997 covering the classical anxiety disorders (Separation anxiety, Social Anxiety and GAD) as well as panic disorder and school phobia was developed for the DSM IV [22]. Its psychometric properties were found to be good as well in a replication study [23] (see also [2, 3, 24]). Muris [25, 26] added some additional items to screen for OCD, specific Phobias (SP) and acute/post-traumatic stress disorder (A/PTSD) as well. The revised scale is called the SCARED-R and its reliability and discriminant validity was studied further in a clinical sample [27]. Muris [27] found a satisfactory discriminant validity both in between the anxiety- and other disorders as well as within the group of anxiety disorders itself. He noted that the SCARED-R had a reasonable capacity to predict specific anxiety disorders. No normative SCARED or SCARED-R data have been published in Sweden. However, in Norway (with a culture close to Sweden) Leikanger [2, 3] used the original SCARED in a sizeable sample of adolescents and has published normative data, as well as data on symptom change across 1 year.

The SCARED-R was translated into Swedish in 2008 for use in a treatment project in paediatric OCD [28]. In the current paper, the utility of the Swedish SCARED/SCARED-R is examined in a clinical diagnostic study [29] and psychometric data are published for the first time. In addition, the diagnostic accuracy is improved as we use KSADS diagnoses improved on through a LEAD process (see below) to validate the SCARED-R and its sub-scales. The advantage with the LEAD process is that additional information that may not be divulged in an interview setting may come to the foreground as the work-up and the treatment unfolds. Moreover, the level of impairment that each disorder entails is assessed not only at the intake assessments, but is supplemented through both structured and more informal contacts with the school that the LEAD procedure may consider.

## Aims

To recommend cut-off scores for the SCARED-R based on ROC curves for each scale/sub-scale, and to investigate their usefulness in a psychiatric outpatient population based on the psychometric properties. The SCARED-Rs concurrent and discriminant validity against the LEAD diagnoses are examined.

## Methods

### Subjects

In all we included 307 CAP outpatients, who consecutively (from January 2010 to March 2013) sought treatment at four different CAP clinics in southern Sweden, in the study. The clinics were the only provider of specialist level care and were situated in average level socio-economic areas for Sweden. Exclusion criterion was the need of interpretation for parents or patient. Forty cases were discarded due to protocol violations (one clinician used leading questions or did not ask both parent and child questions about all symptom areas) or failure of the diagnostician to report data. Another 28 cases were withdrawn due to insufficient additional information in the medical records up until 6 months from intake, a pre-requisite for an adequate LEAD procedure. Data from the remaining n = 239 cases are reported. Mean age of the 239 participants was 12.1 (sd. 3.1, range 6.1–17.8) years old. The observation time that had yielded new information was 1.2 (sd. 0.6) years with a range 0.1–3.1 years. The proportion of children 6–12 years was n = 138 (57, 7%). There were slightly more boys (n = 131, 54.8%) than girls included and boys were slightly younger than girls (11.9 vs. 12.4 years, p = .007). Almost all children had clinically significant disorders, with current Children’s Global Assessment Scale scores (CGAS) for boys just below 50 (m = 48.0, SD = 0.46) and girls 50 (m = 50.0, SD = 0.56). No one had CGAS scores in the non-clinical range of 70 or above.

Further, out of the 239 patients, a SCARED self-rating scale was filled in by 204 patients (boys n = 112/girls n = 92 respectively) and the corresponding parent-rating scale was filled in by 228 parents (boys n = 122/girls n = 106). Hence, numbers will vary somewhat across analyses.

### Measures and Procedures

A comprehensive description of measures and procedures can be found in a previous report on the KSADS by Jarbin [30]. Shortly, the semi-structured interview, the the Schedule for Affective Disorders and Schizophrenia for School-Age Children- Present and Lifetime Version (K-SADS-PL) was used by resident MDs (either CAP specialists or in training to become CAP specialists) following a training program (see [30]). The KSADS interviews with both parents and patients yielded DSM IV diagnoses which were then further evaluated using a “Longitudinal Expert All Data” (LEAD) process commonly viewed as the best proxy to a “gold standard” that can be used to evaluate semi-structured interviews. Through the LEAD process, all information brought in through diagnostic procedures, the level of impairment, and the outcome of treatment across a suitable time period is used [31–33] for the final LEAD diagnosis or diagnoses (see [31] for a detailed description). To be eligible for LEAD, the record should cover at least 6 months of follow-up from the K-SADS-PL and include at least three further visits or significant information from a teacher or an assessment of a senior clinician. In the LEAD procedure, the judge has access to the K-SADS-PL interview as well as subsequent information from the medical records. All these data were retrieved using a structured form. Thus, the re-evaluation of the KSADS diagnoses was systematic and could include oral reports and report forms from teachers and other informants, psychological assessments and the outcome of pharmacological and psychological treatment. For further information about the reliability etc. of this process, see Jarbin [30]. Furthermore, a research assistant asked the patient and parent to fill in the SCARED (as well as some other scales) during one visit.

### Statistics

T-tests or χ^2^ tests were conducted to examine gender differences, differences between diagnoses etc. Receiver operating characteristics (ROC) analyses were conducted to examine the concurrent validity of the SCARED total score and subscales [34]. Generally, the area under the curve (AUC) is judged to represent low accuracy between 0.50 and 0.70, moderate accuracy between 0.70 and 0.90 and >0.90 high accuracy [35]. Agreement between LEAD diagnoses and cut-off for the SCARED scales and sub-scales were studied using the Kappa statistic. Regarding Kappa values a common interpretation is that: Poor agreement = Less than 0.20; Fair agreement = 0.20–0.40; Moderate agreement = 0.40–0.60; Good agreement = 0.60–0.80; Very good agreement = 0.80–1.00. We also conducted series of logistic regression analysis: first, to evaluate the concurrent and discriminant validity of the SCARED subscales vis-à-vis all anxiety diagnoses; second, sequential logistic regression analyses were conducted to examine whether adding an informant (child or parent) would increase how accurately children with a specific anxiety disorder could be identified based on the relevant SCARED subscale.

## Results

### Sample Characteristics

Based on the LEAD procedure, patients displayed a psychiatric disorder pattern that is representative of that seen in CAP-clinics (Table 1) with almost 1/3rd affective disorders, a good 1/3rd anxiety disorders and many with neuropsychiatric disorders (ADHD, tics/Tourette and Autism Spectrum Disorders) (69.3%). In the latter case gender differences were strongly evident for the most prevalent disorder [i.e. ADHD combined type 36.7% boys/17.9% girls χ^2^(2, 12.341 p = .002)], more so than in depressive and anxiety disorders (apart from social phobia), where gender differences were quite small. As the number of patients that had specific anxiety disorders differed, some tests are at risk for type II errors. However, for most disorders, our study provides enough patients to study psychometric properties of the SCARED-R with sub-scales (Table 1).

**Table 1:** The frequency of psychiatric disorders in the outpatient sample

Psychiatric disorders	Boys		Girls		Total	
	N	%	N	%	N	%
Any affective disorder	40	26.7	40	34.2	80^a^	30.0
Anxiety disorder total	48	32.0	50	42.7	98^a^	36.7
Separation anxiety disorder (SAD)	12	8.0	10	8.5	22^a^	8.2
Social phobia (SoP)	6	4.0	13	11.1	19^c^	7.1
Generalized anxiety disorder (GAD)	6	4.0	10	8.5	16^a^	6.0
Anxiety NOS	7	4.7	6	5.1	13^a^	4.9
Obsessive-compulsive disorder	7	4.7	5	4.3	12^a^	4.5
Specific phobia (SP)	26	17.3	17	14.5	43^a^	16.1
Panic disorder (PD)/Agoraphobia	2	1.3	6	5.1	8^a^	3.0
Posttraumatic stress disorder	0	0	2	1.1	2^a^	0.7
Any neurodevelopmental disorder^d^	117	78	68	58.1	185^b^	69.3
Any disruptive behavioural disorder^e^	57	38	36	30.8	93^a^	34.8%

Gender differences: ^a^n.s.; ^b^p < .001; ^c^p = .031; ^d^i.e. ADHD, Autism Spectrum and tics/Tourette’s disorder; ^e^i.e. oppositional-defiant disorder and conduct disorder

### Gender and Informant Differences

Girls scored higher than boys on the SCARED/SCARED-R total scores self-ratings. Such gender differences were less and non-significant in parent ratings, in line with the small gender difference in the prevalence of “any anxiety disorder” (Table 2). This pattern, is evident as well at the level of specific anxiety diagnoses except OCD, where self- and parental ratings were comparable.

**Table 2:** Means, standard deviations and independent t-test as per diagnostic group for self- respectively parent ratings separately for boys and girls

	Child rating				Parent rating			
Scale/subscale (number of items)	All N = 204 M (SD)	Boys N = 112 M (SD)	Girls n = 92 M (SD)	t-test	All N = 228 M (SD)	Boys n = 122 M (SD)	Girls n = 106 M (SD)	t-test
SCARED total score (41 items)	21.33 (12.73)	17.67 (11.99)	25.79 (12.22)^c^	−4.774***	16.09 (12.42)	15.46 (12.30)	16.83 (12.57)^c^	−0.836
SCARED-R total score (69 items)	35.16 (19.89)	29,24 (18,89)^a^	42.37 (18.75)^c^	−4.956***	23.19 (16.72)	22.21 (16,65)^a^	24.34 (16.81)^c^	−0.966
SAD (8)	3.60 (2.95)	2.88 (2.68)	4.49 (3.03)^c^	−4.034***	2.94 (3,06)	2.71 (2.90)	3.21 (3.23)^c^	−1.227
SoP (7)	5.10 (3.36)	4.61 (3.36)^c^	5.71 (3.27)^c^	−2.352*	3,77 (4,03)	3.77 (4.01)^c^	3.77 (4.08)^c^	0.003
GAD (9)	5.51 (4.23)	4.29 (3.91)	7.00 (4.16)^b^	−4.781***	4.79 (4.15)	4.38 (4.05)	5.26 (4.24)^b^	−1.611
SchoP (5)	2.29 (2.14)	1.93 (1.93)	2.74 (2.30)	−2.737**	2.30 (2.29)	2,30 (2,36)	2.30 (2.21)	0.016
OCD (9)	5.21 (3.45)	4.83 (3.37)^a^	5.67 (3.51)^c^	−1.745	2.32 (2.49)	2,41 (2,51)^a^	2.22 (2.48)^c^	0.587
SP (13)	6.64 (4.78)	5.22 (4.13)	8.37 (4.97)^c^	−4.851***	3.99 (3.74)	3.61 (3.62)	4.43 (3.85)^c^	−1.674
PD (13)	4.82 (4.49)	3.96 (4.13)	5.86 (4.71)^c^	−3.061**	2.30 (3.84)	2.30 (3.86)	2.30 (3,84)^c^	−0.006
APTSD (4)	1.98 (2.24)	1.52 (2.06)	2.53 (2.33)^c^	−3.300***	0.79 (1.42)	0.73 (1.36)	0.86 (1.49)^c^	−0.703

SCARED subscales: *SAD* Separation Anxiety Disorder, *SoP* Social phobia, *GAD* Generalized anxiety disorder, *SchoP* School phobia, *OCD* Obsessive-compulsive disorder, *SP* Specific phobia, *PD* Panic disorder, *APTSD* Acute or post-traumatic stress disorder

*p < .05; **p < .01; ***p < .001

Significant differences between child and parent report: a = p < .001; b = p < .01; c = p < .05

Thus, the moderate parent-child agreement (range about r = 0.5 for school phobia and panic disorder to r = 0.37 for GAD, all p < .001) and the particularly low agreement we saw on the OCD-scale (r = 0.21, p = 0.004), may have different causes. It may be caused by girls’ elevated self-ratings but also by boys’ low self-ratings when compared with parental ratings (Table 2). However, boys’ self-ratings varied, and were similar to parents’ ratings for some sub-scales, and even significantly higher than their parents’ ratings on two sub-scales (OCD and SoP) (Table 2).

### Screening Efficiency

First, we conducted a series of ROC analyses to evaluate how efficiently the total scores and subscales would predict the presence of any anxiety disorder and specific ADs (See Table 3). All predictions were significant. The child-reported total scores (SCARED and SCARED-R) predicted the presence of any AD with low accuracy and the parent-reported total scores predicted the presence of any AD with moderate accuracy. The disorder-specific subscales predicted their corresponding disorder with low to moderate accuracy (Table 3).

**Table 3:** Psychometric properties for the SCARED-R versus a LEAD diagnosis of any anxiety disorder and the specific anxiety disorders

SCARED scale/subscale -> LEAD	Report	AUC	P	Cut-off	Sensitivity %	Specificity %	Kappa
SCARED total score → Any anxiety	Child	0.66	<0.001	≥15	84	43	0.23
	Parent	0.72	<0.001	≥14	68	61	0.27
SCARED-R total score → Any anxiety	Child	0.65	<0.001	≥25	84	43	0.23
	Parent	0.72	<0.001	≥34	43	89	0.34
SAD → SAD	Child	0.76	<0.001	≥5	78	70	0.21
	Parent	0.84	<0.001	≥5	79	80	0.32
SoP → SoP	Child	0.85	<0.001	≥8	77	80	0.29
	Parent	0.89	<0.001	≥9	75	88	0.39
GAD → GAD	Child	0.71	0.008	≥6	79	60	0.11
	Parent	0.74	0.002	≥8	56	80	0.17
OCD → OCD	Child	0.84	<0.001	≥10	60	91	0.31
	Parent	0.84	<0.001	≥9	46	97	0.42
SP → SP	Child	0.61	0.043	≥7	68	57	0.13
	Parent	0.68	0.001	≥5	63	70	0.21
PD → PD	Child	0.84	<0.001	≥6	92	69	0.18
	Parent	0.88	<0.001	≥4	83	83	0.28

*AUC* Area under the curve, *SAD* separation anxiety disorder, *SP* specific phobia, *SoP* social phobia, *GAD* generalized anxiety disorder, *PD* panic disorder, *OCD* obsessive-compulsive disorder, *APTSD* acute or post-traumatic stress disorder

Secondly, we selected the most efficient cut-offs to minimize false-positive and false-negative equally by focusing on maximizing efficiency κ(0.5) [36], and evaluated the sensitivity and specificity of these cut-off scores (Table 3). In all instances, the Kappa [κ(0.5)] showed fair agreement between the total scores/subscales and their corresponding diagnosis except that the cut-off values for the GAD subscale had poor agreement with the LEAD GAD diagnosis (Table 3).

Most SCARED/SCARED-R sub-scales for self-ratings showed adequate “Area Under the Curve” (AUC) in ROC analysis (Table 3), ranging from low accuracy for specific phobias (AUC = 0.61, p = 0 043) to a moderate, bordering on high, (AUC = 0.89, p < .001) for parental rating of social anxiety OCD (Table 3). Thus, most cut-off scores that are based on the ROC analyses (based on the point where both sensitivity and specificity are optimal) can be used with confidence, given that the purpose of its use is in line with the way we estimated it. The sensitivity ranges from the lowest (60%) for OCD to the highest (92%) for PD. The corresponding specificity ranged from the lowest (60%) for GAD to the highest (91%) for OCD.

The SCARED and SCARED-R total scores show corresponding adequate AUC levels, with cut-off yielding high sensitivity, low specificity but still fair kappa levels (Table 3).

Thirdly, we also conducted a series of logistic regression analyses to evaluate the concurrent and discriminant validity of each subscale, i.e. verifying whether only the corresponding subscale of the SCARED would be associated with particular LEAD diagnoses (Table 4). The Odds Ratios (OR), based on logistic regression, shows whether a SCARED/SCARED-R sub-scale relates to the LEAD anxiety diagnoses. It shows, for example that for every additional point scored on the child SAD scale or the parent SAD scale, the likelihood increases 1.42 (p = .001), respectively 1.59 (p = .001) times, that a SAD diagnosis will be present. Likewise, the other SCARED scales (e.g. SoP and GAD) were significantly respectively tended to be related to their respective LEAD diagnoses, but the child SoP scale tended to predict a GAD as well but the GAD sub-scale did not predict SoP (see Table 4 for figures). On the other hand, the parent SoP sub-scale discriminated well against both SAD and GAD, but the parental GAD scale increased the likelihood of both a GAD (OR = 1,14, p = .055), a SoP (OR = 1,19, p = .026) and a PD diagnosis (OR = 1,34, p = .018) but decreased the likelihood of a SAD diagnosis (OR = .79, p = .013).

**Table 4:** Convergent/divergent validity of the SCARED child- and parent report versus LEAD diagnoses using logistic regression where the Odds Ratio (OR) refers to the likelihood of a diagnosis for every additional score point on the scale/sub-scale

SCARED-R	LEAD diagnoses					
	SAD OR (95% CI) P	SP OR (95% CI) P	SoP OR (95% CI) P	PD OR (95% CI) P	GAD OR (95% CI) P	OCD OR (95% CI) P
Child-report						
χ^2^, P	20.066, p = .003	5.53 p = .478	33.067, p < .001	13.155, p = .041	19.271, p = .004	16.065, p = .013
SAD	1.50 (1.21, 1.85) < 0.001	0.93 (0.78, 1.11) 0.440	0.96 (0.76, 1.22) 0.746	1.07 (0.76, 1.50) 0.690	1.23 (0.98, 1.54) 0.078	0.95 (0.71, 1.26) 0.716
SP	0.92 (0.80, 1.05) 0.228	1.10 (0.99, 1.23) 0.075	0.93 (0.79, 1.09) 0.347	0.97 (0.78, 1.19) 0.753	0.96 (0.82, 1.11) 0.551	1.09 (0.91, 1.30) 0.336
SoP	1.07 (0.90, 1.28) 0.439	0.92 (0.81, 1.04) 0.190	1.55 (1.25, 1.94) < 0.001	1.05 (0.81, 1.35) 0.731	1.17 (0.96, 1.43) 0.111	0.98 (0.79, 1.21) 0.844
PD	1.01 (0.85, 1.20) 0.905	1.03 (0.91, 1.17) 0.650	1.12 (0.95, 1.33) 0.187	1.18 (0.95, 1.48) 0.142	1.02 (0.86, 1.21) 0.835	0.93 (0.76, 1.14) 0.486
GAD	0.88 (0.72, 1.06) 0.177	0.96 (0.84, 1.11) 0.596	1.09 (0.92, 1.29) 0.314	1.16 (0.89, 1.51) 0.265	1.23 (1.02, 1.49) 0.030	1.05 (0.84, 1.33) 0.655
OCD	1.00 (0.81, 1.23) 0.996	1.04 (0.89, 1.21) 0.633	0.83 (0.66, 1.05) 0.114	0.91 (0.66, 1.26) 0.582	0.84 (0.66, 1.08) 0.170	1.39 (1.08, 1.78) 0.009
Parent-report						
χ^2^, P	42.811, p < .001	11.268, p = .080	43.997, <0.001	19.027, p = .004	16.549, p = .011	24.060, p = .001
SAD	1.79 (1.40, 2.28) < 0.001	0.96 (0.84, 1.11) 0.610	0.78 (0.61, 1.00) 0.054	1.06 (0.79, 1.43) 0.688	1.01 (0.83, 1.22) 0.959	0.86 (0.66, 1.11) 0.237
SP	0.78 (0.62, 097) 0.027*	1.13 (1.01, 1.26) 0.027	1.11 (0.94, 1.32) 0.224	1.00 (0.81, 1.24) 0.987	1.06 (0.90, 1.24) 0.497	1.14 (0.96, 1.35) 0.134
SoP	1.11 (0.96, 1.29) 0.172	1.09 (0.99, 1.19) 0.097	1.47 (1.23, 1.77) < 0.001	0.89 (0.71, 1.13) 0.340	1.12 (0.98, 1.29) 0.092	0.89 (0.74, 1.08) 0.247
PD	1.14 (0.94, 1.37) 0.176	0.99 (0.88, 1.11) 0.874	0.96 (0.83, 1.10) 0.530	1.15 (0.98, 1.35) 0.086	1.03 (0.90, 1.17) 0.667	1.09 (0.93, 1.28) 0.266
GAD	0.78 (0.62, 0.97) 0.28*	1.01 (0.90, 1.12) 0.919	1.21 (1.03, 1.43) 0.022	1.32 (1.02, 1.71) 0.038	1.18 (1.01, 1.37) 0.035	1.02 (0.84, 1.23) 0.861
OCD	0.90 (0.65, 1.26) 0.544	1.00 (0.83, 1.21) 0.975	0.97 (0.75, 1.24) 0.776	1.05 (0.77, 1.43) 0.767	0.88 (0.68, 1.13) 0.308	1.47 (1.12, 1.94) 0.006

*Low SP score = more likelihood of LEAD SAD

For the additional SCARED-R scales, the OCD scales worked best (OR = 1.39, p = 0 009) and (OR = 1.47, p = .006) respectively with limited overlap against other diagnoses. However, both the SP and PD scales were weaker predictors of their “own” diagnosis and related to one other diagnosis each OCD and SAD respectively.

### Adding Parent Information to Child Information (and Vice Versa)

We also evaluated the possible benefit of adding the SCARED parent-report to the child-report (and vice versa) in predicting the presence of LEAD ADs, using a sequential logistic regression analysis, to evaluate whether the SCARED total score and subscales would predict the presence/absence of any anxiety disorder (SAD, SoP, GAD, OCD, SP and, PD). We entered the child-report first and then added the parent-report following which we started out with the parent-report first and then added the child-report. Thus, the unique contribution of each informant to the other was evaluated.

The final results of the logistic regression analyses are shown in Table 5. The goodness-of-fit in testing the prediction of anxiety disorders was good in all cases (Hosmer-Lemeshow p > .05). In single variable models, both the child- and parent-reports of the SCARED-R total score predicted the presence of any anxiety disorder, and ORs were (3.98, 95% CI 1.95, 8.11 and 5.79, 95% CI 2.82, 11.91 respectively) explaining (R^2^) 0.11 and 0.16 respectively proportion of the variation. However, in both instances we observed significant benefits of adding the parent-report to the child report (Δχ^2^_Parent_ = 19.081, p < .001) respectively adding the child-report to the parent-report (Δχ^2^_Child_ = 10.508, p < .001). In the final model both child- and parent-report each contributed significantly to the prediction of any anxiety disorder (see Table 5 for OR and χ^2^).

**Table 5:** Sequential logistic regression to test the effects of child and parent report on the SCARED for the prediction of any anxiety disorder or specific anxiety disorders

LEAD diagnosis	SCARED Scale—Informant	OR (95%)	Wald	Full model χ^2^	Full model adding an extra report χ^2^_a_	R^2^
Any anxiety univariate models	Revised total—child	3.98 (1.95, 8.11)	14.389***	16.543***		0.11
	Revised total—parent	5.79 (2.82, 11.91)	22.844***	25.116***		0.16
Any anxiety multivariate model	Revised total—child	3.23 (1.54, 6.79)	9.551**	35.624***	Δ10.508***	0.22
	Revised total—parent	4.89 (2.33, 10.25)	17.687***	35.624***	Δ19.081***	
Any anxiety univariate model	Total score—child	3.85 (1.88, 7.85)	13.687***	15.678***		0.10
	Total score—parent	2.92 (1.60, 5.32)	12.255***	12.801***		0.09
Any anxiety Multivariate model	Total score—child	3.26 (1.57, 6.77)	10.048**	23.890***	Δ11.089***	0.16
	Total score—parent	2.45 (1.32, 4.56)	8.030**	23.890***	Δ8.212**	
SAD univariate models	SAD – child	7.96 (2.51, 25.26)	12.371***	15.367***		0.16
	SAD—parent	15.59 (4.82, 50.41)	21.042***	26.444***		0.27
SAD Multivariate model	SAD—child	3.18 (0.88, 11.54)	3.096	29.774***	Δ3.330	0.31
	SAD—parent	9.49 (2.67, 33.82)	12.057***	29.774***	Δ14.407***	
SoP univariate models	SoP—child	11.76 (3.59, 38.56)	16.537***	19.787***		0.22
	SoP—parent	20.74 (6.17, 69.77)	23.997***	28.608***		0.31
SoP Multivariate model	SoP—child	6.29 (1.74, 22.75)	7.868**	37.232***	Δ8.624**	0.40
	SoP—parent	12.72 (3.56, 45.44)	15.314***	37.232***	Δ17.445***	
GAD Univariate models	GAD—child	5.45 (1.47, 20.20)	6.435*	7.976**		0.10
	GAD—parent	6.29 (1.99, 19.77)	9.854**	10.318***		0.13
GAD Multivariate model	GAD—child	3.98 (1.04, 15.32)	4.039*	15.043***	Δ4.725*	0.18
	GAD—parent	4.77 (1.47, 15.49)	6.759**	15.043***	Δ7.067**	
OCD Univariate models	OCD—child	14.17 (3.65, 54.93)	14.698***	14.118***		0.21
	OCD—parent	36.60 (7.96, 168.20)	21.406***	19.201***		0.28
OCD Multivariate model	OCD—child	18.18 (3.21, 102.83)	10.758***	31.140***	Δ11.938***	0.44
	OCD—parent	47.74 (7.07, 322.47)	15.734***	31.140***	Δ17.021***	
SP Univariate models	SP—child	3.04 (1.31, 7.02)	6.743**	7.321**		0.06
	SP—parent	3.96 (1.76, 8.93)	11.042***	11.558***		0.10
SP Multivariate model	SP—child	2.17 (0.90, 5.26)	2.963	14.640***	Δ3.082	0.12
	SP—parent	3.17 (1.36, 7.39)	7.085**	14.640***	Δ7.319**	

In the multivariate model, the contribution of the child to the parent report and vice versa are added to the values in the single report from the uni-variate analysis (e.g., parent OR = 4.89 are added to the child OR = 3.89). The Δ19.081 is the difference between the full model χ^2^ 35.624 − 16.543 etc

For the SCARED total score both child-, and parent-report predicted the presence of any anxiety disorder in a single-variable model (OR were 3.85, 95% CI 1.88, 7.85 and 2.92, 95% CI 1.60, 5.32 respectively) with R^2^ 0.10 and 0.09 respectively. In both instances the model improved by adding the other informant (Δχ^2^_Parent_ = 8.212, p < .01 and Δχ^2^_Child_ = 11.089, p < .001) (see Table 5 for OR and χ^2^).

For the SAD subscale both versions were significant in the single variable model (OR = 7.96, 95% CI 2.51, 25.26 and OR = 15.59, 95% CI 4.82, 50.41) with R^2^ 0.16 and 0.27 respectively. However, the model improved only by adding parent-report to the child-report (Δχ^2^_Parent_ = 14.407, p < .001) but not by adding the child-report to the parent-report (Δχ^2^_Child_ = 3.30) (see Table 5 for OR and χ^2^).

For the SoP subscales both child- and parent-report showed significant ORs in the single variable model (OR were 11.76, 95% CI 3.59, 38.56 and 20.74, 95% CI 6.17, 69.77 respectively) with R^2^ 0.22 and 0.31 respectively. Both models improved by adding an extra informant (Δχ^2^_Parent_ = 17.445, p < .001 and Δχ^2^_Child_ = 8.624, p < .01) (see Table 5 for OR and χ^2^).

We found statistically significant associations between the GAD subscales and the presence of GAD both for the child-report and the parent report (OR were 5.45, 95% CI 1.47, 20.20 and 6.29, 95% CI 1.99, 19.77 respectively) with R^2^ 0.10 and 0.13 respectively individually. Both models improved by adding an informant (Δχ^2^_Parent_ = 7.067, p < .01 and χ^2^_Child_ = 4.725, p < .05) (see Table 5 for OR and χ^2^).

Next, we analysed whether the OCD subscales would predict the presence/absence of a LEAD OCD diagnosis, which both the child-report (OR = 14.17, 95% CI 3.65, 54.93, R^2^ = 0.21) and parent-report (OR = 36.60, 95% CI 7.96, 168.20, R^2^ = 0.28) did. In addition, the model improved by adding an extra informant (Δχ^2^_Parent_ = 17.021, p < .001 and Δχ^2^_Child_ = 11.938, p < .001) (see Table 5 for OR and χ^2^).

We also assessed whether the SP subscales would predict the presence or absence of a LEAD SP diagnosis. Individually, both the child-report (OR = 3.04, 95% CI 1.31, 7.02, R2 = 0.06) and the parent-report (OR = 3.96, 95% CI 1.76, 8.93, R^2^ = 0.10) had significant association with the SP diagnosis. We also observed a significantly improved model by adding the parent-report to the child-report (Δχ^2^_Parent_ = 7.319, p < .01). However, adding the child-report to the parent-report did not improve the overall model (Δχ^2^_Child_ = 3.083, n.s.) (see Table 5 for OR and χ^2^).

## Discussion

The study is the first to evaluate the utility of the SCARED-R scale and sub-scales based on the psychometric properties versus the gold-standard called LEAD [31–33]. It can thus be expected to provide the most accurate assessment of its usefulness and validity of its predictions. The population that we used was child-psychiatric outpatients having a level of co-morbidity that can be expected from a general clinical population (Table 1) [37]. Thus, the assessments include the various confounds that the clinician is confronted with in the clinic.

First, the SCARED original scale (i.e. that published by Birmaher [21]) and which assesses the three “classical” child anxiety disorders (separation anxiety disorder, social anxiety disorder and generalized anxiety disorder), and panic disorder could be shown to be a valid measure with regard to each of these four diagnostic entities (kappa values against LEAD ranged from 0.11 to 0.21 for self-ratings and from 0.17 to 0.39 for parental ratings). Moreover, the three classical anxiety disorders and panic disorder SCARED scales showed moderate AUC values. Generally, the area under the curve (AUC) is judged to represent low accuracy between 0.50 and 0.70, moderate accuracy between 0.70 and 0.90 and above 0.90 high accuracy [35].

The cut-off values for these scales rendered acceptable sensitivity and specificity. However, there are caveats, mainly based on the limited agreement between the child and the parent (ranging from 0.37 to 0.48) and the poor agreement against LEAD for GAD (Table 3). Despite this, adding an informant is worthwhile and increases the diagnostic precision (Table 5).

Although, the gender differences may point to a general bias for under-reporting symptoms in boys and over-reporting symptoms in girls, parents’ ratings were not so far above boys’ self-ratings. Possibly, girls’ reports are the bigger problem, and one can speculate whether their scores reflect a different judgment of severity, or if the scale measures “en passant” other phenomena than just anxiety disorders. Despite these caveats, we conclude that the three classical SCARED sub-scales show good psychometric properties in line with previous studies [15] and can be used with confidence using the cut-off values we provide (Table 3).

School phobia is not a DSM IV/5 diagnosis so that its psychometric properties cannot be studied versus an anxiety specific LEAD diagnosis.

The situation with regard to the added subscales in the SCARED-R [27] is similar. Sub-scales for OCD and specific phobia were studied (as A/PTSD had only two cases the psychometric properties are not discussed). These sub-scales were valid measures regarding corresponding two diagnostic entities (kappa values against LEAD were respectively 0.13 and 0.31 for self-ratings and 0.42 and 0.21 for parental ratings). Moreover, these scales showed moderate AUC values, and the cut-off values for these scales rendered acceptable sensitivity and specificity. However, there are caveats, mainly based on the limited agreement between the child and the parent (ranging from 0.12 to 0.47).

Moreover, the gender differences may point to a general bias for under-reporting symptoms in boys and over-reporting symptoms in girls. Despite these caveats, we conclude that the two added SCARED sub-scales show acceptable psychometric properties.

However, a clinician must consider for what purpose the scale is used. The cut-off values described for each scale or sub-scale are mathematical constructs rather than, for example a useful cut-off value for screening. Cut-off values in Table 3 are points on the ROC-curves where the most optimal compromise between sensitivity and specificity is obtained. In situations when screening for anxiety disorders in the clinic, it may be proper to use a lower cut-off score with a higher sensitivity with a concomitant lower specificity. Thus, the purpose must dictate what cut-off score to use. Our data indicate that the SCARED-R is useful as a screen for the classical anxiety disorders, but also for OCD, panic and specific phobias.

In primary/first line child psychiatric care, the two total scores may have some usefulness, e.g. screening for the level of anxiety without wishing to pinpoint specific diagnoses. In such cases, child ratings with a sensitivity of about 80% may be preferable.

Despite the caveats described above, adding the parent as an informant, or vice versa is worthwhile and increases the diagnostic precision (Table 5), contrary to one previous report [15]. However, with regard to SCARED SP respectively SCARED SAD only parent reports added to the child report, while the child report did not increase diagnostic precision to the parental report. In all other instances, both the child and the parent report added to the parent respectively child report, thus increasing diagnostic precision.

Moreover, while using the SCARED-R, the clinician should keep in mind that the properties of this scale are related to the DSM IV anxiety disorders constructs [22], and that these properties may differ from those of DSM-5 diagnoses in some ways.

## Summary

Both parents and children provide unique information, contributing in most diagnostic areas to a correct diagnosis. We urge clinicians to use information from both the child and the parent both when using a scale like the SCARED/SCARED-R and when assessing within an un-structured clinical interview. In most cases, each provides unique information. However, at which items this contribution is most crucial is not known, but could be studied in this sample.

First, even if this is a sizable study, the number of participants in some diagnostic groups were too small, i.e. we had only two patients diagnosed with a post-traumatic stress disorder. Moreover, just eight patients with panic disorder/agoraphobia increases the risk of error. The 12 patients with OCD was as well rather few. However, for the major “classical” anxiety disorders numbers were clearly adequate so that the concurrent and discriminant validity of these measures can be viewed as established.

Secondly, LEAD diagnoses based on enhancing KSADS with systematic diagnosis related information up to 6 months from inclusion, is still at some risk for chance variation in the information that is available within the medical records. As the SCARED was not a base for the LEAD diagnoses, artificially elevated validity measures are excluded.

Thirdly, the data that our study provides needs to be seen within the context of a normative study, for example regarding gender differences. A Norwegian study, i.e. from a culture close to the Swedish culture, of the SCARED showed data that are compatible with our findings [2] and showed gender differences that were quite similar to our findings.

The SCARED/SCARED-R is generally a valid and reliable screening tool. Particularly the classical SCARED has good psychometric properties and can be used with confidence. However, the added sub-scales (i.e., SCARED-R) shows more disparate findings, The OCD scale shows mixed results regarding validity/screening efficiency, which, despite a limited patient base, was statistically significant. Regarding the SP scale, the psychometrics were not as good, despite an adequate number of patients.

The cut-off scores we provide represent a compromise between sensitivity and specificity, optimizing both. However, if a particular use of the SCARED scale presupposes, for example, a higher sensitivity, so that a lower cut-off score is used, then unavoidably specificity will be lower than the figures we provide, and the statistical association with the diagnoses will be changed. In general, but less exactly, the figures for adding child to parent information and vice versa are applicable.
